# Cognitive Biases, Social Influences and Receptivity to Behavioral Interventions in Sustainable Food Choices: Evidence from the Romanian FRAME Pilot Study

**DOI:** 10.3390/foods15213883

**Published:** 2026-10-29

**Authors:** Răzvan-Ionuț Drugă, Kamer-Ainur Aivaz, Cristina Duhnea, Elena Condrea, Ionela Munteanu, Diane-Paula-Corina Vancea, Silvia Ghiță-Mitrescu, Ionuț Antohi

**Affiliations:** 1 Department of General Economy, Faculty of Economic Sciences, Ovidius University of Constanta, 900470 Constanta, Romania; kamer.aivaz@365.univ-ovidius.ro (K.-A.A.); elena.condrea@365.univ-ovidius.ro (E.C.); 2 Department of Finance and Accounting, Faculty of Economic Sciences, Ovidius University of Constanta, 900470 Constanta, Romania; cristinaduhnea@univ-ovidius.ro (C.D.); ionela.munteanu@365.univ-ovidius.ro (I.M.); economics@ovidius-university.net (D.-P.-C.V.); silvia.mitrescu@365.univ-ovidius.ro (S.G.-M.); 3 Department of Business Administration, Faculty of Economic Sciences, Ovidius University of Constanta, 900470 Constanta, Romania; antohi.ionut@365.univ-ovidius.ro

**Keywords:** sustainable food consumption, cognitive biases, perceived cost barriers, social norms, nudge acceptability

## Abstract

This cross-sectional mixed-methods study examines psychological, economic, and social factors associated with sustainable food choices and receptivity to behavioral interventions among adults in Romania. Conducted within the Erasmus+ FRAME project, the study used an online survey of 244 participants recruited through convenience sampling in May 2026. The questionnaire assessed cognitive biases, perceived cost barriers, social influences, information gaps and trust, and receptivity to interventions involving default portion sizes, social-norm messages, and choice-preserving nudges. Quantitative analyses identified positive associations between cognitive biases and perceived cost barriers, social influences and intervention receptivity, and information gaps and trust-related perceptions and intervention receptivity. Overall receptivity was slightly above the neutral midpoint, with greater support for autonomy-preserving nudges and default portion sizes than for plate-finishing norm messages. Qualitative findings highlighted interconnected economic, behavioral, cultural, and informational barriers. The study contributes evidence on how these dimensions coexist in a Romanian sample and identifies differences in the perceived acceptability of behavioral interventions. The findings may inform context-sensitive intervention development and pilot testing, while highlighting the need for representative and experimental research before conclusions about behavioral effectiveness can be drawn.

## 1. Introduction

The global food system is responsible for approximately one-third of anthropogenic greenhouse gas emissions, making dietary choices a critical lever for climate change mitigation [1]. Despite growing awareness of environmental issues and widespread positive attitudes toward sustainability, a persistent gap exists between consumers’ stated values and their actual food purchasing and consumption behaviors [2,3]. This phenomenon, known as the attitude–behavior gap or value–action gap, represents one of the most significant challenges in promoting sustainable food consumption [4].

Romania, as a member of the European Union (EU), is subject to the European Green Deal and Farm to Fork Strategy, which seek to promote healthier and more sustainable food systems [5]. However, sustainable dietary transitions occur within specific economic, institutional, and cultural environments. In Romania, perceived affordability constraints, established food traditions, and the coexistence of traditional and modern food retail environments may shape consumers’ opportunities and motivations to adopt sustainable dietary practices. These circumstances highlight the importance of examining the interrelationships between economic barriers, psychological processes, social influences, and information-related perceptions rather than treating sustainable food consumption as an exclusively individual choice [6].

Behavioral economics and nudge theory provide a framework for understanding how food choices may be influenced through changes in choice architecture without eliminating alternatives [7]. Nudging strategies, including default options, visual cues, portion-size modifications, and social-norm messages, have been examined in relation to healthier eating, food waste reduction, and environmentally sustainable consumption [8,9]. Evidence from systematic reviews and meta-analyses suggests that these interventions can influence food-related decisions, although their effects vary across intervention types and settings [10,11]. Consequently, nudging should be understood as a context-sensitive approach rather than a universally effective behavioral solution.

An important source of variation concerns the social and cultural environment in which interventions are introduced. Food choices are embedded in family practices, shared expectations, social identities, and culturally established eating routines. These influences may affect both the perceived relevance of behavioral messages and consumers’ willingness to accept changes in food-choice environments. Research on sustainable consumption emphasizes the importance of social norms and contextual influences, while evidence on green nudges indicates that intervention characteristics and implementation settings are relevant to their outcomes [12,13]. Social-norm interventions may therefore be received differently depending on whether the communicated behavior is considered credible, desirable, and compatible with existing practices.

Economic conditions constitute another important consideration. Price sensitivity, perceived affordability, and the availability of sustainable alternatives can constrain consumers’ ability to act on pro-environmental preferences. Such constraints are especially relevant when sustainable products are perceived as more expensive or less convenient than conventional alternatives. Recent reviews of green consumption interventions suggest that behavioral approaches must be considered alongside economic and structural conditions rather than as substitutes for them [14]. Accordingly, consumers may express favorable attitudes toward sustainable food choices while remaining reluctant to adopt them when financial or practical barriers are salient.

Psychological characteristics further complicate the relationship between sustainability attitudes and food-related decisions. Habitual purchasing, convenience orientation, environmental values, perceived behavioral control, and trust in sustainability information can shape how consumers evaluate available alternatives. Systematic evidence indicates that sustainable food choices are influenced by interacting motivational, cognitive, and situational factors [13,15]. Moreover, experimental research suggests that reflection on dietary preferences can improve responses to sustainability-oriented nudging, indicating that behavioral interventions may benefit from engaging consumers’ existing motivations rather than relying exclusively on automatic decision-making [16]. These findings also highlight the importance of distinguishing the behavioral effectiveness of an intervention from consumers’ stated receptivity to it.

Despite growing research on sustainable food consumption, several questions remain relevant for understanding the conditions under which behavioral interventions are perceived as acceptable. Existing evidence often examines psychological, economic, social, or informational influences separately, while less attention is given to how these dimensions are associated within the same consumer population. Furthermore, research on the effectiveness of nudges does not necessarily establish whether consumers experiencing different barriers perceive particular interventions as useful or acceptable. This distinction is important because favorable perceptions may facilitate implementation but do not guarantee subsequent behavioral change.

The Romanian context provides an informative setting for examining these relationships. Its combination of perceived affordability concerns, established family food practices, and changing food retail environments offers an opportunity to investigate whether psychological, social, and economic dimensions commonly identified in sustainable consumption research are also interconnected in a specific Central and Eastern European setting. The theoretical relevance of this context therefore extends beyond its relative underrepresentation in the literature: it allows established assumptions concerning consumer autonomy, social influence, perceived costs, and information-related barriers to be examined together.

Against this background, the present study investigates associations among cognitive biases, perceived cost barriers, social influences, information gaps and trust, and receptivity to behavioral interventions in a Romanian convenience sample. It also examines differences in the perceived acceptability of specific intervention approaches and explores participants’ explanations of sustainable food consumption barriers and facilitators. By integrating quantitative associations with qualitative accounts, the study contributes to a more context-sensitive understanding of the attitude–behavior gap and generates empirically grounded questions for future behavioral intervention research. Given its cross-sectional design, the study focuses on perceived barriers and intervention receptivity rather than causal effects or observed behavioral change.

In this study, sustainable food consumption is examined through respondents’ self-reported food-related practices, perceived barriers, and perceptions of interventions intended to support more sustainable choices. The study does not directly quantify the environmental impact of participants’ diets or measure the attitude–behavior gap using observed purchasing or consumption data.

## 2. Literature Review

### 2.1. The Attitude–Behavior Gap in Sustainable Food Consumption

The discrepancy between consumers’ environmental attitudes and their actual purchasing behavior has been extensively documented in the sustainable consumption literature [2,3,4]. Systematic reviews have consistently identified this gap as a central challenge in promoting sustainable food choices [15]. Munro et al. (2023) conducted a comprehensive review of the sustainable attitude–behavior gap in supermarket shopping contexts, identifying psychological, situational, and structural factors that contribute to this disconnect [4]. These factors include cognitive biases, economic constraints, social norms, information asymmetries, and choice architecture.

Fernqvist et al. (2024) synthesized findings from multiple systematic reviews on food choice, highlighting the complexity of decision-making processes involving sensory preferences, health considerations, convenience, price, and environmental concerns [15]. Their analysis revealed that environmental sustainability often ranks lower than taste, price, and convenience in actual purchasing decisions. This suggests that interventions must consider multiple barriers simultaneously to promote sustainable food consumption.

The Theory of Planned Behavior (TPB) posits that behavior is determined by intentions, which are shaped by attitudes, subjective norms, and perceived behavioral control [17]. However, the TPB has been criticized for its limited ability to predict actual behavior, particularly in the sustainability domain, where the intention–behavior gap remains substantial [2]. This limitation has prompted researchers to explore additional psychological constructs, including habits, cognitive biases, and automatic decision-making processes [18].

### 2.2. Cognitive Biases and Habitual Food Choices

Cognitive biases—systematic deviations from rational decision-making—play a significant role in food choice [18,19]. Habitual behavior, in particular, represents a form of automatic decision-making that bypasses deliberative cognitive processes [20]. When food choices become habitual, consumers rely on established routines and heuristics rather than actively evaluating the sustainability implications of their decisions. This automaticity can perpetuate unsustainable consumption patterns even among individuals who hold pro-environmental attitudes.

The status quo bias, which reflects a preference for maintaining current behaviors, is particularly relevant to food consumption [21]. Consumers tend to repeat familiar food choices, shop at the same stores, and follow established meal patterns. This bias is reinforced by the cognitive effort required to evaluate new information, compare alternatives, and change established routines, potentially creating inertia that prevents the adoption of more environmentally friendly dietary patterns.

Convenience orientation reflects consumers’ preference for food options that minimize time and effort [22]. In food environments characterized by time scarcity and competing demands, convenience often takes precedence over sustainability considerations. These cognitive and habitual factors are therefore relevant to understanding why consumers may continue established dietary practices despite expressing favorable attitudes toward sustainable consumption.

In the present study, the Cognitive Bias construct operationalizes habitual and convenience-oriented decision tendencies, together with the perceived cognitive effort involved in considering sustainability. These indicators do not capture the full range of cognitive biases and should be distinguished from external practical constraints, such as limited time, product availability, and affordability.

### 2.3. Perceived Cost Barriers

Economic factors represent one of the most consistently cited barriers to sustainable food consumption [23,24]. Sustainable food products—including organic, locally sourced, fair trade, and environmentally certified items—may carry price premiums that make them less accessible to price-sensitive consumers. In Romania, where household incomes remain below the EU average and food expenditure represents a significant portion of household budgets, price sensitivity is particularly relevant.

The perception that sustainable food is expensive can deter consumers even when price differences are modest or when sustainable alternatives, such as seasonal produce or plant-based proteins, may be more affordable than conventional options [25]. This perception can be shaped by limited product availability, inadequate price transparency, and marketing strategies that position sustainable products as premium offerings.

Willingness to pay (WTP) for sustainable food attributes varies across countries, product categories, and consumer segments [25]. While some consumers demonstrate high WTP for sustainability attributes, others express conditional willingness to purchase sustainable products when prices are comparable to conventional alternatives. These findings suggest that perceived affordability and economic constraints should be considered alongside psychological factors when examining sustainable food choices.

### 2.4. Social Influences on Food Choice

Food consumption is inherently social, shaped by family traditions, cultural norms, peer influences, and social identity [26]. Social influence operates through informational influence, normative influence, and identification with valued social groups [27].

Descriptive norms—perceptions of what others typically do—and injunctive norms—perceptions of what others approve or disapprove—can influence food choices [9]. Communicating descriptive norms may encourage sustainable consumption, particularly when individuals identify with the reference group. However, norm-based interventions may also produce unintended effects if messages are perceived as irrelevant or communicate that unsustainable behavior is common.

Family and cultural traditions play an important role in food choice, particularly in contexts where traditional cuisine and home cooking remain prevalent. Sustainable food choices may sometimes conflict with family preferences or cultural identity. Conversely, practices such as seasonal eating, home preservation, and food waste reduction can align with sustainability principles. These considerations highlight the relevance of social and cultural influences when examining consumers’ receptivity to behavioral interventions.

### 2.5. Information Gaps and Trust in Sustainability Labels

Effective decision-making requires accessible, credible, and comprehensible information. However, the proliferation of sustainability labels, certifications, and claims has created an information-rich but potentially confusing environment [28,29]. Potter et al. (2021) conducted a systematic review of environmental sustainability labels, finding that labels can influence food selection and purchasing, although their effectiveness depends on label design, consumer understanding, and trust in certifying bodies [29].

Information overload—the cognitive burden of processing excessive information—can lead to decision difficulties or reliance on simplified heuristics [18]. When consumers encounter multiple competing labels, such as organic, fair trade, carbon neutral, or locally sourced, they may struggle to compare alternatives or prioritize sustainability attributes. Trust in sustainability certifications is shaped by perceptions of certifying organizations’ credibility, independence, and rigor [28]. Concerns about greenwashing may further undermine confidence in sustainability claims. Consequently, information clarity and trust represent important dimensions of sustainable food decision-making and may also be relevant to consumers’ perceptions of interventions designed to simplify choices.

### 2.6. Behavioral Interventions and Nudging

Nudge theory, popularized by Thaler and Sunstein, proposes that subtle changes to choice architecture can influence behavior while preserving freedom of choice [7]. Unlike traditional policy tools such as taxes, subsidies, or regulations, nudges work by making certain options more salient, convenient, or attractive without forbidding alternatives [30]. Nudges have been applied to promote healthier eating, reduce food waste, and encourage sustainable consumption [8,9].

Common nudging techniques in food contexts include default options, portion size manipulation, strategic placement of products, descriptive norm messaging, and visual cues [31,32]. Pandey et al. (2023) conducted a meta-analysis of nudging interventions in university canteens, finding significant effects on sustainable food selection, although effect sizes varied by intervention type and context [9].

The acceptability of nudges depends on their perceived intrusiveness and alignment with personal autonomy [30]. Interventions that preserve choice and are perceived as helpful rather than manipulative may be more acceptable. Balancing effectiveness, acceptability, and ethical considerations therefore remains a key challenge in nudge design.

Kwasny et al. (2022) reviewed interventions to reduce meat consumption, identifying education, persuasion, incentivization, and environmental restructuring as common approaches [33]. Their findings highlight the potential value of interventions that address both deliberative and automatic decision-making processes [33,34]. Nevertheless, evidence of behavioral effectiveness should be distinguished from consumers’ stated receptivity to interventions, as favorable perceptions do not necessarily translate into behavioral change.

### 2.7. The Romanian Food System Context

Romania’s food system reflects its historical, economic, and cultural context. Following the transition from communism in 1989, Romania experienced significant economic restructuring, including the privatization of agriculture and the entry of multinational food retailers. This transformation occurred against the background of a formerly centralized economy, in which state control over production and investment had constrained market-based competition and individual investment opportunities [35]. Despite modernization, traditional food practices, including home cooking, home gardening, and direct procurement from local producers, remain relevant.

Perceived affordability is a relevant consideration when examining sustainable food choices in the Romanian context. Consumers who regard sustainable products as more expensive than conventional alternatives may experience difficulties reconciling sustainability preferences with household budget priorities. The present study examines these perceived economic barriers at the respondent level rather than drawing conclusions about national income or expenditure patterns.

The Romanian retail landscape combines modern supermarkets and hypermarkets with traditional markets and small neighborhood stores. These different retail environments may create variation in the availability, accessibility, and perceived affordability of sustainable food options.

Cultural factors also shape Romanian food consumption. Traditional practices involving seasonal ingredients, home-cooked meals, and food preservation may align with sustainability principles, while changing lifestyles and consumption patterns can create new challenges. Understanding these influences is relevant to examining how economic, psychological, and social dimensions of sustainable food consumption are associated in the Romanian context.

Rather than representing a contribution solely because it is underrepresented in existing research, the Romanian context provides an opportunity to examine the relationships among established behavioral dimensions within a specific economic and cultural environment. In particular, the coexistence of perceived affordability concerns, habitual food choices, family influences, and information-related challenges offers a relevant setting for investigating consumers’ receptivity to sustainable food interventions.

### 2.8. Research Questions

The literature reviewed above indicates that sustainable food consumption is shaped by interconnected psychological, economic, social, and informational factors. Although these dimensions have been extensively examined individually, their associations with consumers’ receptivity to behavioral interventions warrant further investigation in specific consumption contexts.

Accordingly, the present study aims to examine the relationships among cognitive biases, perceived cost barriers, social influences, information gaps and trust, and receptivity to behavioral interventions among Romanian respondents. The analysis is guided by four research questions:

**RQ1.**  *What is the relationship between cognitive biases, including habitual and convenience-oriented food choices, and perceived cost barriers to sustainable food consumption?*

**RQ2.**  *How are social influences associated with consumers’ receptivity to behavioral interventions designed to promote sustainable food choices?*

**RQ3.**  *How are information gaps and trust-related perceptions associated with receptivity to behavioral interventions?*

**RQ4.**  *To what extent do respondents express receptivity to different behavioral interventions, and what qualitative barriers and facilitators help explain their perceptions?*

By addressing these questions through quantitative and qualitative evidence, this study seeks to provide a context-sensitive understanding of sustainable food consumption and behavioral intervention receptivity in Romania, while recognizing that the cross-sectional design does not permit causal conclusions or direct assessment of intervention effectiveness.

## 3. Materials and Methods

### 3.1. Study Design and Context

This study employed a cross-sectional mixed-methods design combining quantitative survey data with qualitative open-ended responses. The research was conducted as part of the Romanian pilot of the FRAME (Food Resilience through Architecturally Modelled Environments) project, an Erasmus+ Alliances for Innovation initiative involving four countries: Romania, Greece, Türkiye, and Italy. The FRAME project aims to enhance food system resilience through evidence-based behavioral interventions, stakeholder engagement, and capacity building.

The Romanian pilot was designed to examine the psychological, social, and economic factors influencing sustainable food consumption among Romanian consumers, food business representatives, and policymakers. The study focused on five key constructs identified through literature review and stakeholder consultation: cognitive biases, perceived cost barriers, social influences, information gaps and trust, and receptivity to behavioral interventions.

### 3.2. Participants and Sampling

Participants were recruited through non-probability convenience sampling using online channels, including social media, email lists, and partner organizations’ networks. Eligibility criteria included being 18 years or older and residing in Romania. The final sample comprised 244 respondents who completed the survey in May 2026. The demographic characteristics of the sample are summarized in Table 1.

The sample included three stakeholder groups: consumers (*n* = 221, 90.6%), food business representatives (*n* = 11, 4.5%), and policymakers (*n* = 12, 4.9%). Given the predominance of consumers in the sample, quantitative analyses focused on the full sample while acknowledging the consumer-centric composition. Qualitative analyses considered stakeholder perspectives where relevant.

The sample size reflected the exploratory scope and practical recruitment conditions of the Romanian FRAME pilot rather than a probability-based sampling design intended to produce nationally representative estimates. The three stakeholder groups were included to capture perspectives from consumers and other food-system actors. Quantitative analyses used the pooled sample to examine the measured constructs across respondents; however, because consumers represented 90.6% of participants, the results primarily reflect consumer responses. The small numbers of business representatives and policymakers do not support reliable comparisons between stakeholder groups.

Regarding territorial coverage, the questionnaire recorded respondents’ residential environment (urban or rural) but did not collect information on their county or region of residence. Therefore, the geographical distribution of the sample across Romania cannot be established. Of the 244 respondents, 208 (85.2%) reported living in urban areas and 36 (14.8%) in rural areas. These characteristics, together with the non-probability recruitment approach, limit the geographical generalizability of the findings.

### 3.3. Instrument Development

The survey instrument consisted of three main sections: (1) demographic and background questions, (2) 25 Likert-scale items measuring five constructs, and (3) open-ended qualitative questions.

Demographic variables included gender, age, education level, household size, residential area (urban/rural), income adequacy, employment status, cooking frequency, and primary role (consumer, business representative, or policymaker).

The quantitative component comprised five constructs, each assessed using five items on a 5-point Likert scale (1 = Totally disagree, 5 = Totally agree): (1) Cognitive Bias (CB) assessed habitual food choices, automaticity, convenience orientation, and perceived effort required to consider sustainability; (2) Perceived Cost Barriers (PCB) measured perceptions of sustainable food affordability, price prioritization, and conditional willingness to pay; (3) Social Influences (SI) evaluated interpersonal influence, conformity, family and cultural influences, conflicts with traditions, and social pressure; (4) Information Gaps & Trust (IGT) assessed understanding of sustainability labels, information overload, confusion, trust in certifications, and confidence in identifying sustainable products; and (5) Behavioral Interventions (BI) measured receptivity to specific behavioral approaches, including default portion sizes, descriptive norm messages, injunctive norms, perceived social concern about food waste, and autonomy-preserving nudges.

The questionnaire items were developed by the FRAME project team specifically for the Romanian pilot study, drawing on constructs identified in the sustainable food consumption literature. The instrument comprised 25 items organized into five constructs, as described above. The questionnaire was administered in Romanian. The wording of the items is presented in Table 2 to support transparency and interpretation of the measures.

Qualitative questions asked respondents to describe, in their own words, (1) barriers to sustainable food consumption, (2) factors that facilitate sustainable choices, (3) the role of information and labels, (4) social and cultural influences, and (5) their willingness to adopt sustainable food practices. These open-ended questions provided contextual insights that complemented the quantitative data.

### 3.4. Data Collection Procedure

Data were collected online using a secure survey platform during May 2026. The survey link was distributed through multiple channels to maximize reach and diversity. Participants accessed the survey voluntarily and could withdraw at any time. The survey took approximately 15–20 min to complete.

The questionnaire was configured with mandatory responses, preventing participants from proceeding to subsequent questions or sections without answering all required items. Consequently, all 244 questionnaires included in the final analytical sample were fully completed and contained no missing responses to the required questions. The final dataset therefore comprised 244 valid and complete questionnaires. The number of participants who may have discontinued the survey before submission was not available.

### 3.5. Ethical Considerations

The study received ethical approval from the Bioethics Committee of Ovidius University of Constanța (Approval No. 5097/05.05.2026). All participants provided informed consent before beginning the survey. The consent form explained the study’s purpose, procedures, risks, benefits, confidentiality measures, and participants’ right to withdraw without penalty.

Data were collected anonymously, with no personally identifiable information recorded. Survey responses were stored securely and accessible only to the research team. Participants were informed that aggregated findings would be published in academic outlets and disseminated to project stakeholders.

### 3.6. Data Analysis

Quantitative analysis was conducted using SPSS version 28.0. Descriptive statistics (means, standard deviations, skewness, and kurtosis) were calculated for all items and scale scores. Internal consistency reliability was assessed using Cronbach’s alpha for each construct.

Normality assumptions were evaluated through skewness and kurtosis values, with absolute values below 2.0 considered acceptable for parametric analyses. Pearson correlation coefficients were calculated to examine the associations between cognitive biases and perceived cost barriers (RQ1), social influences and the Behavioral Interventions score (RQ2), and information gaps and trust-related perceptions and the Behavioral Interventions score (RQ3). A one-sample *t*-test compared the mean Behavioral Interventions score with the neutral midpoint (3.0), while item-level one-sample *t*-tests examined differences between individual item means and the neutral midpoint (RQ4). These items captured related but distinct dimensions, including perceived usefulness of interventions, reported motivation, and perceptions of social norms.

Effect sizes were reported using Cohen’s d for *t*-tests and Pearson’s r for correlations. Statistical significance was set at α = 0.05, and two-tailed tests were used for all reported analyses. To assess the precision of the estimates, 95% confidence intervals were reported for the correlations and mean differences. Confidence intervals for Pearson correlations were estimated using Fisher’s z transformation. The direction, magnitude, and uncertainty of the observed associations and mean differences were considered when interpreting the results. Cronbach’s alpha was interpreted as an indicator of internal consistency rather than as evidence of unidimensionality or construct validity. Particular caution was applied to the Behavioral Interventions and Information Gaps & Trust constructs because their items capture related but potentially distinct dimensions. The item-level comparisons were exploratory and were interpreted primarily in terms of the direction and magnitude of the observed differences, rather than as independent confirmatory tests. No formal adjustment for multiple comparisons was applied; consequently, the reported item-level *p*-values should be interpreted with caution. The dimensional structure of the composite measures was not independently established through factor analysis, and formal diagnostic assessments of linearity and influential observations were not documented. These limitations should be considered when interpreting the correlation results.

To assess the stability of the principal findings, a consumer-only sensitivity analysis was conducted. The three Pearson correlations corresponding to RQ1–RQ3 and the one-sample *t*-test corresponding to RQ4 were repeated using only respondents who identified as consumers (*n* = 221). The resulting estimates were compared with those obtained from the full sample (*N* = 244).

Qualitative analysis employed thematic analysis following Braun and Clarke’s six-phase approach: familiarization with the data, generating initial codes, searching for themes, reviewing themes, defining and naming themes, and producing the report. Qualitative responses were examined thematically to identify recurring ideas relevant to the study’s research questions. The analysis was interpretive and exploratory; no formal intercoder agreement statistic is reported. Themes were identified inductively from the data while remaining informed by the study’s theoretical framework. Representative quotations were selected to illustrate each theme.

The qualitative dataset comprised all 228 available text responses collected through the survey’s open-ended questions from the overall sample of 244 respondents. No additional sampling of the available text responses was undertaken. As individual respondents could provide multiple text responses, the 228 qualitative responses do not represent 228 distinct participants. The unit of qualitative analysis was the individual text response rather than the respondent.

Where stakeholder affiliation was identifiable, qualitative responses were considered in relation to the three stakeholder groups (consumers, business representatives, and policymakers) to identify potentially different perspectives. These observations were subsequently considered alongside the quantitative findings to provide contextual insights into perceived barriers, facilitators, and behavioral intentions.

## 4. Results

### 4.1. Sample Characteristics

Table 1 presents the demographic characteristics of the sample (*N* = 244). The sample was predominantly female (69.7%), urban (85.2%), highly educated (62.7% postgraduate degree), and employed full-time (78.3%). The largest age group was 45–54 years (34.0%), followed by 18–24 years (18.9%) and 35–44 years (18.9%). Most respondents lived in households of two to four persons (80.4%), with three-person households being most common (31.6%).

Regarding economic status, 63.9% reported that their income comfortably covers expenses, 32.8% indicated their income barely covers expenses, and 3.3% stated their income does not cover expenses. Cooking frequency was high, with 39.8% cooking almost every day and 33.6% cooking three to four times per week. The vast majority of respondents identified as consumers (90.6%), with small proportions of food business representatives (4.5%) and policymakers (4.9%).

### 4.2. Descriptive Statistics and Scale Reliability

Table 2 presents descriptive statistics for all 25 survey items. Mean scores ranged from 1.92 (Item 15: “I feel social pressure to eat certain foods”) to 3.59 (Item 16: “I understand sustainability labels”). Standard deviations ranged from 1.00 to 1.30, indicating moderate variability in responses.

Within the Cognitive Bias construct, Item 2 (“My food choices are mostly habitual”) had the highest mean (M = 3.27, SD = 1.10), while Item 4 (“Convenience matters more than environmental impact”) had the lowest (M = 2.39, SD = 1.12). For Perceived Cost Barriers, Item 9 (“Eating sustainably costs more money”) had the highest mean (M = 3.42, SD = 1.07), and Item 7 (“Price matters more than sustainability when I buy food”) had the lowest (M = 2.59, SD = 1.09).

In the Social Influences construct, Item 13 (“Family or culture influences what I eat”) showed the highest mean (M = 3.14, SD = 1.20), while Item 15 had the lowest mean across all items (M = 1.92, SD = 1.00). For Information Gaps & Trust, Item 16 had the highest mean overall (M = 3.59, SD = 1.11), while Item 17 (“There are too many labels to trust”) had a mean of 2.80 (SD = 1.07).

Within Behavioral Interventions, Item 25 (“I find behavioral nudges useful if they do not limit my choice”) had the highest mean (M = 3.56, SD = 1.07), followed by Item 21 (“Making small portions the default option in restaurants reduces my food waste”) (M = 3.40, SD = 1.22). Item 22 (“Messages such as ‘Most people finish their plates’ motivate me”) had the lowest mean in this construct (M = 2.48, SD = 1.08).

Table 3 presents scale reliability statistics. Cronbach’s alpha coefficients ranged from 0.686 (Behavioral Interventions) to 0.850 (Perceived Cost Barriers), indicating acceptable to good internal consistency. All constructs except for Behavioral Interventions exceeded the conventional threshold of α = 0.70. The slightly lower alpha for Behavioral Interventions (0.686) is acceptable given the construct’s breadth, encompassing diverse intervention types.

Skewness values ranged from −0.607 (Information Gaps & Trust) to 0.215 (Perceived Cost Barriers), and kurtosis values ranged from −0.583 (Perceived Cost Barriers) to 1.090 (Information Gaps & Trust). All values fell within acceptable ranges (|skewness| < 2, |kurtosis| < 3), supporting the use of parametric statistical tests.

### 4.3. Associations Among Behavioral, Economic, Social and Informational Dimensions

Table 4 summarizes the principal associations among the examined constructs and the overall level of receptivity to behavioral interventions. The results are presented according to the research questions, with emphasis on the direction and magnitude of the associations and the difference from the neutral midpoint.

Pearson correlation analyses identified positive associations among the examined dimensions. The strongest association was observed between cognitive biases and perceived cost barriers (r = 0.517, 95% CI [0.419, 0.603], *p* < 0.001), indicating that respondents reporting stronger habitual and convenience-oriented food choices also tended to perceive greater economic barriers to sustainable consumption.

Social influences were positively associated with the composite Behavioral Interventions score (r = 0.303, 95% CI [0.185, 0.412], *p* < 0.001). This association was smaller in magnitude, suggesting that participants reporting greater interpersonal and normative influences also tended to express more favorable overall perceptions of behavioral interventions.

Information gaps and trust-related perceptions were also positively associated with the Behavioral Interventions score (r = 0.493, 95% CI [0.392, 0.583], *p* < 0.001). Given that the Information Gaps & Trust scale combines items addressing understanding, confusion, confidence, and trust, this result should be interpreted as an association between the composite construct and intervention-related perceptions rather than as evidence that information deficits alone increase receptivity. The interpretation of this association also depends on the coding direction of the individual scale items.

The overall Behavioral Interventions score was slightly above the neutral midpoint (M = 3.13, t(243) = 2.65, *p* = 0.009, Cohen’s d = 0.17), indicating a statistically significant but small difference. The corresponding 95% confidence interval for the mean difference was [0.033, 0.224]. Given the heterogeneity of the items included in this scale, the composite score should be interpreted as a general indicator of intervention-related perceptions rather than a uniform measure of receptivity to all intervention types.

These findings describe associations and self-reported perceptions within the sample. They do not establish causal relationships or demonstrate the behavioral effectiveness of the interventions examined.

A consumer-only sensitivity analysis (*n* = 221) was conducted to assess the stability of the principal findings. The results were similar to those obtained from the full sample (*N* = 244). The correlation between Cognitive Bias and Perceived Cost Barriers was r = 0.502 (compared with r = 0.517 in the full sample), the correlation between Social Influences and Behavioral Interventions was r = 0.283 (compared with r = 0.303), and the correlation between Information Gaps & Trust and Behavioral Interventions was r = 0.510 (compared with r = 0.493). All three correlations remained statistically significant (two-tailed *p* < 0.001). The mean Behavioral Interventions score also remained slightly above the neutral midpoint (M = 3.12, t(220) = 2.310, two-tailed *p* = 0.022, Cohen’s d = 0.155, 95% CI for the mean difference [0.017, 0.218]), compared with M = 3.13, t(243) = 2.65, *p* = 0.009, and Cohen’s d = 0.17 in the full sample. These findings suggest that the principal results were not substantially altered by excluding business representatives and policymakers. Nevertheless, the sensitivity analysis does not address the limitations associated with convenience sampling or establish national representativeness.

### 4.4. Item-Level Analysis of Behavioral Interventions

To examine differences in respondents’ perceptions of specific behavioral interventions and related social norms, one-sample *t*-tests were conducted for each Behavioral Interventions item against the neutral midpoint (3.0). The results are presented in Table 5. Two-tailed tests were used to assess whether item means differed significantly from the midpoint, irrespective of direction.

Three items had mean scores significantly above the neutral midpoint, although they represented different aspects of intervention-related perceptions and social influence.

Item 25 (“I find behavioral nudges useful if they do not limit my choice”) had the highest mean (M = 3.56, t(243) = 8.167, *p* < 0.001, mean difference = 0.557, 95% CI [0.423, 0.692]), indicating relatively favorable perceptions of interventions that preserve consumer autonomy.

Item 21 (“Making small portions the default option in restaurants reduces my food waste”) also had a mean significantly above the midpoint (M = 3.40, t(243) = 5.122, *p* < 0.001, mean difference = 0.402, 95% CI [0.250, 0.560]). This finding suggests that respondents generally perceived default portion sizes as potentially useful for reducing their food waste. However, the study did not measure actual food waste reduction.

Item 24 (“People around me care about reducing food waste”) showed an above-neutral mean (M = 3.26, t(243) = 3.620, *p* < 0.001, mean difference = 0.262, 95% CI [0.120, 0.405]). Unlike Items 21 and 25, this item measures perceived social concern about food waste rather than direct receptivity to a behavioral intervention.

One item had a mean significantly below the neutral midpoint. Item 22 (“Messages such as ‘Most people finish their plates’ motivate me”) received a mean of 2.48 (t(243) = −7.568, *p* < 0.001, mean difference = −0.525, 95% CI [−0.661, −0.388]). This result indicates relatively low self-reported motivation in response to this specific plate-finishing message. It should not be generalized to all descriptive norm interventions, as their acceptability and effectiveness may depend on message content and context.

Item 23 (“People think leaving food on the plate is wrong”) did not differ significantly from the neutral midpoint (M = 2.95, t(243) = −0.689, *p* = 0.491, mean difference = −0.053, 95% CI [−0.206, 0.100]). This result provides no clear evidence that respondents, on average, perceived strong social disapproval related to leaving food on the plate.

Overall, the item-level findings indicate meaningful variation in respondents’ perceptions of behavioral interventions and food waste-related social norms. Choice-preserving nudges and default portion sizes received relatively favorable evaluations, whereas the specific plate-finishing message elicited lower reported motivation. These differences underscore the importance of examining intervention approaches individually rather than relying exclusively on an overall composite score. Because the measures capture self-reported perceptions, they cannot establish whether the interventions would produce corresponding changes in actual food choices or food waste.

### 4.5. Qualitative Findings

Thematic analysis of all 228 available text responses collected through the survey’s open-ended questions identified six major themes: Behavioral Barriers, Economic Barriers, Social Influences, Information Gaps and Trust, Sustainable Food Practices, and Behavioral Intentions. As individual respondents could provide multiple text responses, the qualitative dataset represents 228 units of textual analysis rather than 228 distinct participants.

#### 4.5.1. Theme 1: Behavioral Barriers

Respondents frequently cited behavioral and psychological barriers to sustainable food consumption, including lack of time, convenience orientation, established habits, and the cognitive effort required to make sustainable choices. Many described tension between knowing what is environmentally responsible and acting on that knowledge in daily life.

*“Since I have limited free time, I make choices about cooking products in a hurry, without taking sustainability into consideration.”*

(Participant 57, Female, 55+, Consumer)

Time scarcity emerged as a dominant sub-theme, with respondents explaining that busy schedules, work demands, and family responsibilities leave little time for deliberative food shopping or meal preparation. Convenience foods, despite being less sustainable, fit better into time-constrained lifestyles.

Habitual behavior was also prominent, with respondents acknowledging that they tend to repeat familiar food choices without actively considering sustainability. Breaking established routines was described as requiring conscious effort and motivation that is difficult to sustain amid competing priorities.

#### 4.5.2. Theme 2: Economic Barriers

Economic constraints were a recurring theme mentioned as a barrier across all stakeholder groups. Consumers emphasized that sustainable food products carry price premiums that make them inaccessible or require trade-offs with other household expenses. Many expressed conditional willingness to purchase sustainable food if prices were comparable to conventional alternatives.

*“The high price is the main obstacle; most of the time, I have to prioritize my monthly budget over sustainable products, which are considerably more expensive.”*

(Participant 74, Female, 45–54, Consumer)

Business representatives highlighted supply chain costs, including raw material prices, logistics, and certification expenses, as barriers to offering affordable sustainable products. They noted that consumer price sensitivity limits their ability to pass costs to customers.

Policymakers acknowledged affordability challenges and discussed the need for subsidies, tax incentives, or other fiscal measures to make sustainable food more accessible. Several mentioned the tension between promoting sustainability and ensuring food security for low-income populations.

#### 4.5.3. Theme 3: Social Influences

Social and cultural factors emerged as important influences on food choice, operating through family traditions, peer behavior, and cultural identity. Respondents described food consumption as deeply embedded in social relationships and cultural practices.

*“The family is the environment where eating behavior is formed.”*

(Participant 8, Female, 35–44, Consumer)

Family preferences and childhood food habits were frequently mentioned as shaping current food choices. Some respondents noted that sustainable food choices could conflict with family members’ preferences or cultural traditions, creating household tension. Others described family as a source of support for sustainable practices, particularly when multiple family members shared environmental values.

Cultural traditions, particularly around holidays and celebrations, were described as both barriers and facilitators. Traditional Romanian cuisine emphasizes seasonal, local ingredients and home cooking, which align with sustainability. However, certain traditional foods may conflict with environmental goals.

#### 4.5.4. Theme 4: Information Gaps and Trust

Respondents expressed mixed views about sustainability information and labels. While many acknowledged that labels can be useful, concerns about clarity, complexity, standardization, and credibility were widespread.

*“I perceive sustainability labels as useful, but sometimes difficult to understand or compare across products.”*

(Participant 1, Female, 45–54, Consumer)

Information overload was a common complaint, with respondents describing confusion when faced with multiple competing labels and certifications. The proliferation of labels was seen as making comparison difficult rather than facilitating informed choice.

Trust issues centered on concerns about greenwashing and the credibility of certifying organizations. Some respondents expressed skepticism about whether labeled products are genuinely more sustainable or whether labels are primarily marketing tools. Calls for independent verification, transparent criteria, and enforcement against false claims were common.

#### 4.5.5. Theme 5: Sustainable Food Practices

Many respondents described current sustainable food practices they engage in, including purchasing local and seasonal produce, home cooking, meal planning, food waste reduction, minimal packaging, recycling, and choosing organic products when affordable.

*“I try to maintain a balance and make the most sustainable choices possible by choosing local, seasonal foods and products with minimal packaging.”*

(Participant 114, Female, 35–44, Consumer)

Local and seasonal purchasing was the most frequently mentioned practice, often linked to shopping at farmers’ markets or directly from producers. Respondents valued freshness, quality, and supporting local economies in addition to environmental benefits.

Food waste reduction strategies included meal planning, proper food storage, using leftovers creatively, and composting. Some respondents mentioned that minimizing waste was motivated by both environmental concerns and economic considerations (avoiding wasted money).

#### 4.5.6. Theme 6: Behavioral Intentions

Respondents expressed varying levels of willingness to adopt or expand sustainable food practices. Many described conditional intentions, contingent on factors such as price, availability, convenience, and information.

*“The most effective approaches are combinations of measures that make sustainable choices easier, more visible, and more affordable.”*

(Participant 87, Female, 35–44, Policymaker)

Active information-seeking was mentioned by some respondents who described efforts to learn about sustainability, read labels, and research products. These individuals expressed frustration when information was unavailable or unreliable.

Support for multi-component interventions was evident, with respondents suggesting that effective promotion of sustainable consumption requires addressing multiple barriers simultaneously—economic, informational, and structural. Some respondents expressed skepticism about their ability to make a difference through individual choices, citing the need for systemic change and corporate responsibility.

## 5. Discussion

### 5.1. Interrelated Cognitive and Economic Barriers

The positive association between cognitive biases and perceived cost barriers (r = 0.517) suggests that habitual and convenience-oriented food choices coexist with stronger perceptions of financial constraints in the present sample. This finding is consistent with research emphasizing that sustainable food decisions are shaped by interacting psychological and situational factors rather than by environmental attitudes alone. Munro et al. [4] identified multiple mechanisms contributing to the sustainable attitude–behavior gap, while Fernqvist et al. [15] highlighted the importance of price, convenience, and established preferences in everyday food choices.

The present findings extend this discussion by documenting an association between cognitive and perceived economic barriers within the same Romanian sample. One possible interpretation is that consumers who rely on established routines may be less inclined to investigate affordable sustainable alternatives. Conversely, perceived financial constraints may reinforce familiar purchasing patterns. However, these mechanisms remain hypothetical because the cross-sectional design cannot establish their direction or causality.

Recent evidence further suggests that interventions promoting sustainable consumption should account for both behavioral and structural constraints [14]. Accordingly, strategies aimed solely at increasing sustainability awareness may be insufficient when consumers perceive sustainable options as financially inaccessible. The qualitative findings reinforce this interpretation, as participants frequently described time limitations, convenience needs, and affordability concerns as interconnected aspects of everyday food decisions.

The contribution of this finding is therefore not the identification of cost or habit as previously unknown barriers, but the demonstration that these dimensions are meaningfully associated within the investigated sample. This provides a rationale for future studies examining whether reducing cognitive effort and perceived financial barriers simultaneously produces stronger behavioral outcomes than addressing either dimension independently.

### 5.2. Social Influences and the Contextual Relevance of Normative Messages

The positive association between social influences and intervention receptivity (r = 0.303) is consistent with theoretical perspectives emphasizing the role of social expectations and reference groups in sustainable consumption [27,34]. However, the item-level findings indicate that general receptivity to behavioral interventions should not be equated with acceptance of every norm-based message.

Respondents reported relatively favorable perceptions of social concern about food waste (M = 3.26), whereas the statement suggesting that most people finish their plates received a substantially lower rating (M = 2.48). This contrast suggests that the content and framing of normative information may be particularly important. The broader literature indicates that sustainable consumption interventions operate within social and cultural environments that influence the perceived relevance and acceptability of behavioral messages [12,13].

Several interpretations are possible. Plate-finishing messages may be considered less personally relevant, may conflict with portion-control preferences, or may be perceived as placing inappropriate pressure on individuals. Nevertheless, the present data do not directly measure these mechanisms, and they should therefore be treated as explanations requiring further investigation.

Qualitative responses emphasized the importance of family practices, childhood experiences, and cultural traditions in shaping food choices. These observations suggest that the relevance of social influence in the Romanian sample may depend less on explicit conformity pressures and more on established interpersonal and household practices. This distinction is potentially informative for future research examining how culturally embedded food routines interact with the acceptability of sustainability-oriented messages.

### 5.3. Information-Related Perceptions, Trust, and Intervention Receptivity

The association between Information Gaps and Trust and Behavioral Interventions (r = 0.493) indicates that respondents’ information-related perceptions were meaningfully associated with their stated receptivity to behavioral interventions. This finding is relevant to previous research identifying sustainability information as an important but sometimes difficult-to-use resource in food decision-making.

Potter et al. [29] demonstrated that environmental sustainability labels can influence food selection and purchasing, while their usefulness depends on how information is communicated and understood. Grunert et al. [28] similarly emphasized consumer motivation, understanding, and use of sustainability labels.

The present study adds evidence concerning the relationship between a broader information-and-trust construct and perceived intervention receptivity. Respondents reported relatively high confidence in understanding sustainability labels, while qualitative accounts also revealed confusion, perceived information overload, and concerns about credibility. These findings suggest that consumers may simultaneously value sustainability information and experience difficulties evaluating its reliability or practical relevance.

An important qualification concerns the interpretation of the composite measure. Because the scale includes both positively and negatively framed information-related items, the observed correlation should not automatically be interpreted as demonstrating that greater information deficits increase receptivity to nudges. Such a conclusion would require confirmation of item coding, dimensionality, and potentially separate analyses of information clarity, confusion, and trust.

The findings nevertheless support further investigation of how information environments interact with behavioral intervention acceptability. They also suggest that transparent, comprehensible sustainability information and choice-preserving behavioral interventions may be complementary approaches, although their combined effectiveness remains to be tested experimentally.

### 5.4. Differential Receptivity to Behavioral Interventions

Overall receptivity to behavioral interventions was slightly above the neutral midpoint (M = 3.13), but the small effect size (d = 0.17) indicates that this difference should not be overstated. More informative than the overall score was the variation across individual intervention items.

Autonomy-preserving nudges received the highest rating (M = 3.56), followed by default portion sizes (M = 3.40). In contrast, plate-finishing descriptive norm messages received a below-neutral rating. These differences suggest that participants distinguished between interventions perceived as facilitating choices and messages that may be experienced as less relevant or acceptable.

This pattern is consistent with the broader literature on nudge acceptability and ethical choice architecture [7,30]. It also complements evidence that food-related nudges can produce heterogeneous outcomes across intervention types and settings [9,10,11]. Experimental research further indicates that engaging consumers’ preferences and reflective processes may strengthen sustainable dietary choices [16].

However, the present findings concern stated receptivity and perceived usefulness, not experimentally observed changes in food consumption. Therefore, the relatively favorable ratings of autonomy-preserving nudges and default portions should be interpreted as indications of potential acceptability rather than evidence of superior behavioral effectiveness.

The main contribution lies in identifying differences in perceived intervention acceptability within a Romanian sample and relating these perceptions to broader cognitive, social, and informational dimensions. These results refine the assumption that behavioral interventions can be considered a homogeneous category and support the need for intervention-specific evaluation.

### 5.5. Theoretical Contribution and Contextual Implications

Taken together, the findings contribute to sustainable consumption research in three ways.

First, they provide evidence that cognitive and perceived economic barriers are associated within the investigated sample, supporting an integrated rather than isolated understanding of the constraints surrounding sustainable food choices.

Second, they demonstrate that social influences and information-related perceptions are associated with receptivity to behavioral interventions, while also showing that receptivity differs substantially across intervention types. This distinction helps connect the literature on the attitude–behavior gap with research on behavioral intervention acceptability.

Third, the Romanian setting provides an opportunity to examine established theoretical relationships within a food consumption environment characterized, in participants’ accounts, by family traditions, affordability concerns, and mixed perceptions of sustainability information. Rather than claiming that these characteristics are unique to Romania, the study illustrates how established behavioral dimensions may coexist and interact in a particular Central and Eastern European setting.

These contributions should be interpreted within the study’s methodological boundaries. The convenience sample was predominantly female, urban, and highly educated, and the research measured self-reported perceptions at one point in time. Consequently, the findings cannot establish national prevalence, cross-cultural differences, causal mechanisms, or actual intervention effectiveness. Their primary value lies in generating contextually grounded evidence and identifying relationships suitable for confirmation in representative, longitudinal, and experimental research.

### 5.6. Implications for Policy and Practice

The findings suggest several directions for developing and evaluating sustainable food consumption interventions in Romania. Given the cross-sectional design and reliance on self-reported perceptions, these implications should be understood as priorities for intervention development and pilot testing rather than as evidence of demonstrated policy effectiveness.

**Address perceived affordability alongside cognitive barriers.** The positive association between cognitive biases and perceived cost barriers, together with qualitative accounts of financial and time constraints, suggests that sustainable food initiatives should consider affordability and convenience simultaneously. Price-related measures, clearer value-for-money information, and improved accessibility of sustainable alternatives could be evaluated alongside simplified choice environments. This integrated approach is consistent with research emphasizing the interaction between psychological and structural influences on sustainable consumption [4,14,15].

**Improve the clarity and credibility of sustainability information.** The association between information-related perceptions and intervention receptivity, combined with qualitative concerns about confusing labels and greenwashing, supports further attention to accessible and trustworthy sustainability communication. Existing evidence highlights the relevance of label comprehension and consumer trust [28,29]. Policymakers and food businesses could therefore pilot clearer labeling formats, transparent certification information, and communication strategies designed to reduce unnecessary cognitive effort.

**Prioritize the evaluation of autonomy-preserving interventions.** Respondents expressed relatively favorable perceptions of choice-preserving nudges and default portion sizes. These findings suggest that such approaches may be suitable candidates for pilot testing in restaurants, institutional canteens, and food retail environments. However, favorable perceptions should not be interpreted as evidence of actual food waste reduction or dietary change. Randomized field experiments would be necessary to assess their effectiveness and potential unintended consequences [7,9,30].

**Adapt social-norm messages to the target population.** The contrasting ratings of social concern about food waste and plate-finishing messages indicate that normative communication should be evaluated carefully before implementation. Interventions should use credible, contextually relevant messages and avoid assumptions that a particular norm will be equally acceptable across consumer groups. The literature on social influence and sustainable behavior provides a rationale for such context-sensitive approaches [12,27,34].

**Engage households and local food communities.** Qualitative accounts identified family traditions and shared food practices as relevant influences on sustainable consumption. Community-based activities, household-oriented educational initiatives, and communication emphasizing feasible sustainable practices could therefore be explored. Their effectiveness should be assessed empirically rather than assumed on the basis of cultural relevance alone.

**Use phased implementation and rigorous evaluation.** The variation in stated receptivity across interventions highlights the need for small-scale pilots before broader policy adoption. Future evaluations should include observed food choices, purchasing behavior, food waste quantities, distributional effects, and intervention acceptability. Representative sampling and experimental or longitudinal designs would help establish whether the associations identified in this study translate into meaningful behavioral outcomes.

Overall, the findings support the development of context-sensitive interventions that consider economic constraints, cognitive effort, information quality, social practices, and consumer autonomy. Their policy relevance lies primarily in identifying promising intervention targets and evaluation priorities rather than demonstrating the effectiveness of specific measures.

### 5.7. Limitations

Several limitations should be acknowledged. First, the convenience sampling approach limits generalizability. The sample was predominantly female, urban, highly educated, and employed, which may not represent the broader Romanian population. Rural consumers, lower-income households, and less-educated individuals are underrepresented, yet these groups may face distinct barriers to sustainable consumption. Consequently, the findings should be interpreted as sample-specific and exploratory and should not be generalized to the Romanian population.

Second, self-report measures are subject to social desirability bias, potentially inflating reported environmental concern and receptivity to interventions. Behavioral measures (e.g., actual food purchases) would provide a more direct assessment of sustainable consumption, though such data were not feasible within the project’s scope.

Third, the cross-sectional design precludes causal inference. As the analyses focused on associations, the directionality of relationships cannot be definitively established. Longitudinal or experimental designs would be needed to test causal mechanisms.

Fourth, the Behavioral Interventions scale showed acceptable but relatively low internal consistency (α = 0.686), reflecting the diversity of intervention types assessed. Future research could develop separate scales for different nudge categories (e.g., defaults, norms, salience) to enable more fine-grained analysis of self-reported intervention receptivity.

An additional limitation concerns construct validity and potential common-method bias. Although internal consistency was assessed using Cronbach’s alpha, this does not establish that each scale measures a single underlying dimension. In particular, the Behavioral Interventions and Information Gaps & Trust constructs include conceptually heterogeneous items, which may affect the interpretation of their composite scores. Furthermore, because the principal variables were measured through the same self-report questionnaire, shared response tendencies and common-method variance may have influenced the observed associations. Future studies should assess the dimensional structure of the measures and incorporate complementary behavioral or independently collected data.

Finally, the study was conducted in a single country, limiting cross-cultural generalizability. While the Romanian context provides valuable insights relevant to Central and Eastern Europe, the findings may not apply to countries with different economic conditions, cultural norms, or food systems.

## 6. Conclusions

This study provides evidence on the psychological, social, and economic dimensions associated with sustainable food consumption in a Romanian sample. The findings indicate that cognitive biases, perceived cost barriers, social influences, and information-related perceptions are relevant to understanding sustainable dietary choices and receptivity to behavioral interventions. Respondents demonstrated modest receptivity to behavioral interventions, particularly those that preserve autonomy and simplify decision-making, while expressing lower receptivity to certain norm-based messages.

The positive correlation between cognitive biases and perceived cost barriers (r = 0.517) highlights the association between psychological and perceived economic obstacles. This finding suggests that future interventions should consider both the cognitive effort required to identify sustainable options and the real or perceived economic costs of such choices. The positive association between information gaps and trust-related perceptions and receptivity to behavioral interventions (r = 0.493) further suggests that information-related factors warrant consideration when designing and evaluating interventions. However, the cross-sectional data do not establish the direction of these relationships or the effectiveness of particular interventions.

The qualitative findings underscore the importance of family and cultural influences, economic constraints, and the need for credible, standardized sustainability information. Respondents described sustainable practices such as local and seasonal purchasing, home cooking, and food waste reduction, while also identifying barriers related to time, money, and information. Willingness to expand sustainable consumption was frequently conditional on addressing these barriers.

From a theoretical perspective, this study contributes to the literature on the attitude–behavior gap by examining associations among psychological, social, economic, and informational dimensions within a Romanian sample. The findings support the relevance of an integrated perspective combining insights from behavioral economics, social psychology, and consumer research. They also highlight the importance of distinguishing consumers’ stated receptivity to behavioral interventions from their demonstrated behavioral effectiveness.

From a practical perspective, this study identifies potential directions for Romanian policymakers, food businesses, and civil society organizations. Measures addressing perceived affordability, simplified and standardized labeling, autonomy-preserving nudges, family-oriented initiatives, and approaches combining information provision with choice architecture modifications merit further development and empirical evaluation. Their effectiveness cannot be established from the present findings alone.

The FRAME project, through its multi-country, multi-stakeholder approach, provides a framework for collaborative research and intervention design. Future research should extend this work through longitudinal designs to assess behavioral change over time, experimental studies to test specific interventions, and comparative analyses across the four FRAME countries (Romania, Greece, Türkiye, and Italy) to identify context-specific and potentially shared factors.

As Romania continues to develop its food system within the framework of EU sustainability policies, interventions that respect consumer autonomy, address economic constraints, and consider cultural practices may contribute to more sustainable food choices. This study provides a foundation for further investigation by identifying associations among relevant behavioral dimensions and highlighting intervention approaches that respondents perceive as relatively acceptable.

## Figures and Tables

**Table 1:** Sample demographic characteristics (*N* = 244).

Variable		Frequency (*n*)	% of Sample
**Gender**	Female	170	69.7%
	Male	74	30.3%
**Age**	18–24	46	18.9%
	25–34	30	12.3%
	35–44	46	18.9%
	45–54	83	34.0%
	55+	39	16.0%
**Education**	High School	17	7.0%
	Bachelor’s Degree	74	30.3%
	Postgraduate Degree	153	62.7%
**Household Size**	Alone	26	10.7%
	2 persons	58	23.8%
	3 persons	77	31.6%
	4 persons	61	25.0%
	5+ persons	22	9.0%
**Residential Area**	Urban	208	85.2%
	Rural	36	14.8%
**Income** **Adequacy**	Does not cover expenses	8	3.3%
	Barely covers expenses	80	32.8%
	Comfortably covers expenses	156	63.9%
**Employment Status**	Full-time	191	78.3%
	Part-time	7	2.9%
	Student	27	11.1%
	Unemployed	6	2.5%
	Unpaid domestic work	13	5.3%
**Cooking** **Frequency**	Never	9	3.7%
	1–2 times/week	56	23.0%
	3–4 times/week	82	33.6%
	Almost every day	97	39.8%
**Primary Role**	Consumer	221	90.6%
	Business Representative	11	4.5%
	Policymaker	12	4.9%

**Table 2:** Descriptive statistics for survey items (*N* = 244).

Constructs	Items	M	SD
**CB**	1. I choose the same foods without thinking about sustainability	2.66	1.11
	2. My food choices are mostly habitual	3.27	1.10
	3. I prefer quick, easy foods even if less sustainable	2.60	1.17
	4. Convenience matters more than environmental impact	2.39	1.12
	5. Thinking about sustainability takes too much effort	2.46	1.03
**PCB**	6. Sustainable food is too expensive for me	2.62	1.06
	7. Price matters more than sustainability when I buy food	2.59	1.09
	8. I would buy sustainable food if it were cheaper	3.26	1.16
	9. Eating sustainably costs more money	3.42	1.07
	10. Higher prices stop me from choosing sustainable food	2.88	1.14
**SI**	11. Important people influence my food choices	2.85	1.30
	12. I choose sustainable food when others do	2.46	1.05
	13. Family or culture influences what I eat	3.14	1.20
	14. Sustainable food conflicts with my traditions	2.07	1.03
	15. I feel social pressure to eat certain foods	1.92	1.00
**IGT**	16. I understand sustainability labels	3.59	1.11
	17. There are too many labels to trust	2.80	1.07
	18. I find sustainability information on food products confusing	2.82	1.02
	19. I trust sustainability certifications on food products	3.13	1.04
	20. I feel confident identifying genuinely sustainable food products	3.08	1.01
**BI**	21. Making small portions the default option in restaurants reduces my food waste	3.40	1.22
	22. Messages such as “Most people finish their plates” motivate me	2.48	1.08
	23. People think leaving food on the plate is wrong	2.95	1.21
	24. People around me care about reducing food waste	3.26	1.13
	25. I find behavioral nudges useful if they do not limit my choice	3.56	1.07

*Note. All items were assessed on a 5-point Likert scale ranging from 1 (Totally disagree) to 5 (Totally agree), with 3 representing the neutral mid-point. CB = Cognitive Bias; PCB = Perceived Cost Barriers; SI = Social Influences; IGT = Information Gaps & Trust; BI = Behavioral Interventions; M = Mean; SD = Standard Deviation.*

**Table 3:** Scale reliability and distribution statistics (*N* = 244).

Constructs	Cronbach Alpha	Skewness	Kurtosis
Cognitive Bias	0.833	0.174	−0.275
Perceived Cost Barriers	0.850	0.215	−0.583
Social Influences	0.735	0.036	−0.486
Information Gaps & Trust	0.759	−0.607	1.090
Behavioral Interventions	0.686	−0.352	0.508

**Table 4:** Associations and overall receptivity to behavioral interventions (*N* = 244).

Research Question	Statistical Test	Results	95% CI	*p*-Value
RQ1: Cognitive Bias and Perceived Cost Barriers (CB–PCB)	Pearson correlation	r = 0.517	[0.419, 0.603]	<0.001
RQ2: Social Influences and Behavioral Interventions (SI–BI)	Pearson correlation	r = 0.303	[0.185, 0.412]	<0.001
RQ3: Information Gaps & Trust and Behavioral Interventions (IGT–BI)	Pearson correlation	r = 0.493	[0.392, 0.583]	<0.001
RQ4: Overall Behavioral Interventions score compared with the neutral midpoint (3.0)	One-sample *t*-test	M = 3.13; t(243) = 2.65; d = 0.17	[0.033, 0.224]	0.009

*Note. CB = Cognitive Bias; PCB = Perceived Cost Barriers; SI = Social Influences; IGT = Information Gaps & Trust; BI = Behavioral Interventions.*

**Table 5:** Item-level analysis of behavioral interventions (*N* = 244).

Behavioral Intervention Item	M	SD	t(243)	*p*-Value	Mean Difference	95% CI	Interpretation
21. Default portion sizes	3.40	1.22	5.122	<0.001	0.402	[0.250, 0.560]	Above-neutral perceived usefulness
22. Most people finish their plates message	2.48	1.08	−7.568	<0.001	−0.525	[−0.661, −0.388]	Below-neutral reported motivation
23. Leaving food on the plate is wrong	2.95	1.21	−0.689	0.491	−0.053	[−0.206, 0.100]	No statistically significant difference
24. People around me care about reducing food waste	3.26	1.13	3.620	<0.001	0.262	[0.120, 0.405]	Above-neutral perceived social concern
25. Nudges that preserve freedom of choice	3.56	1.07	8.167	<0.001	0.557	[0.423, 0.692]	Above-neutral perceived usefulness; highest mean

*Note. M = mean; SD = standard deviation; CI = confidence interval. All tests compare item means with the neutral midpoint (3.0) using two-tailed one-sample t-tests. Positive mean differences indicate scores above the midpoint, whereas negative differences indicate scores below it. Items assess related but distinct dimensions, including perceived usefulness, reported motivation, and perceptions of social norms.*

## Data Availability

The data presented in this study are available on request from the corresponding author. The data are not publicly available due to privacy and ethical restrictions.

## Abbreviations

The following abbreviations are used in this manuscript:

FRAME	Food Resilience through Architecturally Modelled Environments
TPB	Theory of Planned Behavior
EU	European Union
WTP	Willingness to pay
CB	Cognitive Bias
PCB	Perceived Cost Barriers
SI	Social Influences
IGT	Information Gaps & Trust
BI	Behavioral Interventions
M	Mean
SD	Standard Deviation
RQ	Research Question
CI	Confidence Interval
